# T Lymphocytes in Patients With Nijmegen Breakage Syndrome Demonstrate Features of Exhaustion and Senescence in Flow Cytometric Evaluation of Maturation Pathway

**DOI:** 10.3389/fimmu.2020.01319

**Published:** 2020-06-30

**Authors:** Barbara Piatosa, Beata Wolska-Kuśnierz, Katarzyna Tkaczyk, Edyta Heropolitanska-Pliszka, Urszula Grycuk, Anna Wakulinska, Hanna Gregorek

**Affiliations:** ^1^Histocompatibility Laboratory, Children's Memorial Health Institute, Warsaw, Poland; ^2^Department of Immunology, Children's Memorial Health Institute, Warsaw, Poland; ^3^Department of Oncology, Children's Memorial Health Institute, Warsaw, Poland; ^4^Department of Microbiology and Clinical Immunology, Children's Memorial Health Institute, Warsaw, Poland

**Keywords:** Nijmegen Breakage Syndrome, T lymphocyte maturation, flow cytometry, primary immune deficiency, immune senescence, immune exhaustion

## Abstract

Patients with Nijmegen Breakage Syndrome (NBS) suffer from recurrent infections due to humoral and cellular immune deficiency. Despite low number of T lymphocytes and their maturation defect, the clinical manifestations of cell-mediated deficiency are not as severe as in case of patients with other types of combined immune deficiencies and similar T cell lymphopenia. In this study, multicolor flow cytometry was used for evaluation of peripheral T lymphocyte maturation according to the currently known differentiation pathway, in 46 patients with genetically confirmed NBS and 46 sex and age-matched controls. Evaluation of differential expression of CD27, CD31, CD45RA, CD95, and CD197 revealed existence of cell subsets so far not described in NBS patients. Although recent thymic emigrants and naïve T lymphocyte cell populations were significantly lower, the generation of antigen-primed T cells was similar or even greater in NBS patients than in healthy controls. Moreover, the senescent and exhausted T cell populations defined by expression of CD57, KLRG1, and PD1 were more numerous than in healthy people. Although this hypothesis needs further investigations, such properties might be related to an increased susceptibility to malignancy and milder clinical course than expected in view of T cell lymphopenia in patients with NBS.

## Introduction

Nijmegen Breakage Syndrome (NBS) (MIM #251260) is a rare autosomal recessive disease belonging to a group of chromosomal instability disorders. The disease is caused by mutations in *NBN* gene (MIM #602667) encoding nibrin. The defect leads to defective response to DNA double strand break repair occurring both physiologically and in response to ionizing radiation and radical-producing agents (1–6). The principal clinical manifestations of the syndrome include progressive microcephaly, dysmorphic facial features, mild growth retardation, mild-to-moderate intellectual disability, and an increased predisposition to malignancies (7–10).

Due to humoral and cellular immune deficiency (11–14) patients with NBS suffer from recurrent infections. Low concentration of serum immunoglobulins and/or inadequate specific antibody response (13), are caused by general B cell lymphopenia (11, 15–17) and/or lower frequency of switched memory B-cells (18). Severe impairment in T-cell dependent antigen response and features of defective cellular immunity have been attributed to T cell lymphopenia and defective T lymphocyte maturation (13, 18, 19).

This prospective study was initiated in attempt to describe peripheral T lymphocyte maturation profile in patients with NBS according to the currently known differentiation pathway (20).

## Patients and Methods

Peripheral EDTA-K2 anticoagulated blood samples were collected between November 2016 and December 2018 from 46 patients with common Slavic 657del5 mutation in nibrin (21), and from 46 healthy subjects, with the same female-to-male ratio as in the study group. Detailed clinical data were collected at the time of patient's (or healthy control's) visit in the outpatient department. None of the patients was treated for malignancy or demonstrated other features of lymphoproliferation at their enrollment into the study. In case of previous malignancy, the interval between initiation of the study and the end of treatment associated with remission was at least 2 years. All healthy controls have been sex and age-matched and met additional requirement of smallest possible deviation from the patient's age. They were also free from infections and have not been vaccinated recently.

Distribution of basic lymphocyte populations, including T, B, NK, as well as T helper and cytotoxic lymphocytes, were determined by flow cytometry using the lyse-no-wash approach and Multitest six-color cocktails of antibodies with Trucount tubes, to determine absolute cell counts of respective cell populations (Becton Dickinson, cat. no. 644611) (Table 1). Antibody manufacturer's instructions were followed during the staining procedure. At least 15,000 events were acquired to BD FACSCanto II flow cytometer, with lymphocyte gate based on CD45 expression and side scatter characteristics. Lyse-no-wash settings for the FACS Canto Clinical software were used without any custom modification. Briefly, 50 μl aliquots of blood were incubated with optimally titered antibodies for 15 min in room temperature. The incubation was followed by erythrocyte lysis using 0.45 ml of BD FACSLysing Solution (Becton Dickinson, cat. no. 349202) diluted according to the manufacturer's instructions. Definition of basic lymphocyte subsets, i.e., T, B, NK, CD4, and CD8 T lymphocytes was performed according to standard procedures (22). Absolute numbers of individual cell subsets were calculated based on proportion of the respective cell subpopulation and absolute lymphocyte count (22).

**Table 1:** Six-color antibody panels for composition of the T cell compartment analysis.

**Tube**	**FITC**	**PE**	**PerCP**	**APC**	**APC/Cy7**	**PE/Cy7**
Trucount	CD3 (SK7)^a^	CD16/CD56 (B73.1)^a^/(NCAM16.2)^a^	CD45 (2D1)^a^	CD19 (SJ25C1)^a^	CD8 (SK1)^a^	CD4 (SK3)^a^
1	CD45RO (UCHL1)^b^	CD31 (WM59)^b^	CD3 (SK7)^a^	CD4 (SK3)^a^	CD8 (SK1)^a^	CD45RA (HI100)^b^
2	CD27 (L128)^a^	CD197 (150503)^b^	CD3 (SK7)^a^	CD4 (SK3)^a^	CD8 (SK1)^a^	CD45RA (HI100)^b^
3	CD27 (L128)^a^	CD8 (SK1)^a^	CD3 (SK7)^a^	CD95 (DX2)^a^	CD4 (SK3)^a^	CD45RA (HI100)^b^
4	KLRG1 (SA231A2)^c^	CD8 (SK1)^a^	CD3 (SK7)^a^	CD57 (HCD57)^c^	CD4 (SK3)^a^	CD279 (EH12.1)^b^

*Clones are shown between brackets, manufactures by characters*.

a *BD Biosciences, San Jose, CA, USA*.

b *Pharmingen, San Diego, CA, USA*.

c *Biolegend, San Diego, CA, USA*.

**Figure 1:**
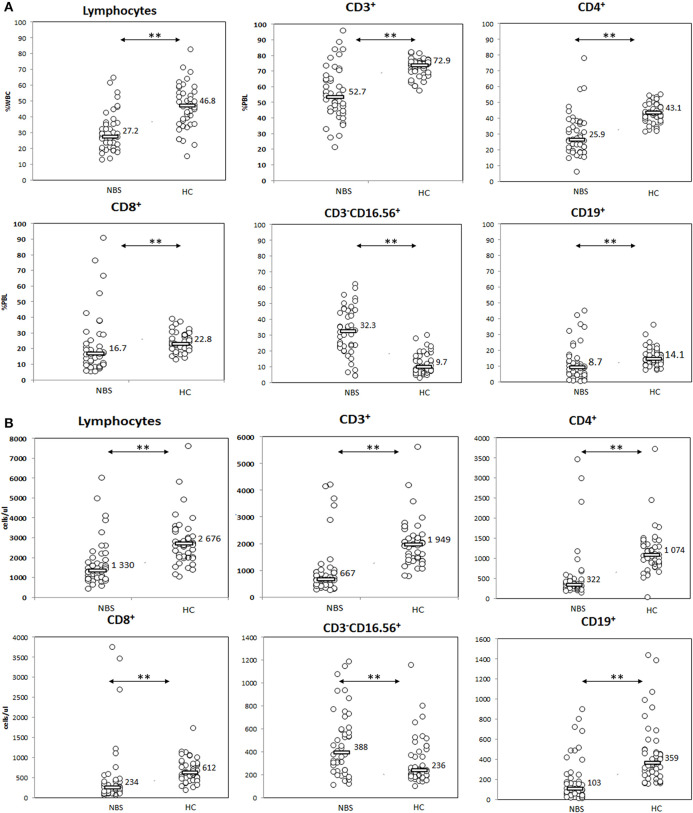
Evaluation of basic lymphocyte subsets. Patients with NBS demonstrated significantly smaller general population of lymphocytes, with disturbed distribution of T (CD3^+^), T CD4^+^ (CD3^+^CD4^+^), T CD8^+^ (CD3^+^CD8^+^), NK (CD3^−^CD16^+^CD56^+^), and B (CD19^+^) lymphocyte subsets in comparison to healthy controls. Individual results in respective study cohorts are presented as circles. Median values are presented as bars with numerical values. Statistical significance: ** <0.01. **(A)** Relative counts and **(B)** absolute counts.

Peripheral T lymphocyte maturation profile was analyzed according to the currently known differentiation pathway, using six-color cocktails of mouse fluorochrome-associated monoclonal antibodies specific for human receptors and differential expression of CD27, CD31, CD45RA, CCR7 (CD197), and CD95 (23) (details on monoclonal antibodies are presented in Table 1). Co-expression of CD31 and CD45RA was used to identify recent thymic emigrants among CD4^+^ lymphocytes and a population of naïve T CD8^+^ lymphocytes including also recently emigrating cells from the thymus. All remaining cell subsets have been identified both within CD4^+^ and CD8^+^ T lymphocyte populations. Naïve population (TN) has been identified as CD27^+^CD45RA^+^CD197^+^, memory T lymphocytes with stem cell-like properties (TSCM) as CD27^+^CD45RA^+^CD95^+^, central memory (TCM) as CD27^+^CD45RA^−^CD197^+^, effector memory (TEM) as CD27^+^CD45RA^−^CD197^−^, terminal effector memory expressing RA (TEMRA), as CD27^−^CD45RA^+^CD95^+^, including low- (L-TEMRA, CD27^+^CD45RA^+^CD197^−^ and high-differentiated H-TEMRA, CD27^−^CD45RA^+^CD197^−^), and late effector memory/terminally differentiated subsets (TD), as CD27^−^CD45RA^−^CD197. We also analyzed features of immunosenescence manifested by expression of CD57 and KLRG1 (24), as well as features of exhaustion associated with expression of PD1 (CD279) (25, 26). Briefly, 100 μl of peripheral blood samples were incubated with an adequate amount of antibodies for 15 min in darkness in room temperature. The sample was then lyzed with BD FACSLysing solution (Becton Dickinson), washed twice with wash buffer (PBS+0.1% sodium azide), and after suspending in wash buffer—acquired into appropriately calibrated BD FACS Canto II cytometer and analyzed with BD Facs Diva v.7 software. Gating strategy applied throughout the study is presented on Figure 1. The same approach was applied for both CD4^+^ and CD8^+^ T lymphocytes. Patient data were compared with results obtained in healthy controls collected during the study, and the differences were analyzed with Mann-Whitney and Fischer's exact tests.

The study was approved by the Bioethical Committee at the Children's Memorial Health Institute, Warsaw (Poland) and carried according to Helsinki Declaration. Written consent for participation was obtained from all patients older than 16 years, and parents or legal guardians in case of patients younger than 16 years.

## Results

The study group included 46 NBS patients aged 0.6–38.7 years (median 12.1 year), with female to male ratio 27:19, and 46 healthy sex-matched controls at similar age (0.6–39.8 year, median 11.9, *p* = NS).

**Figure 2:**
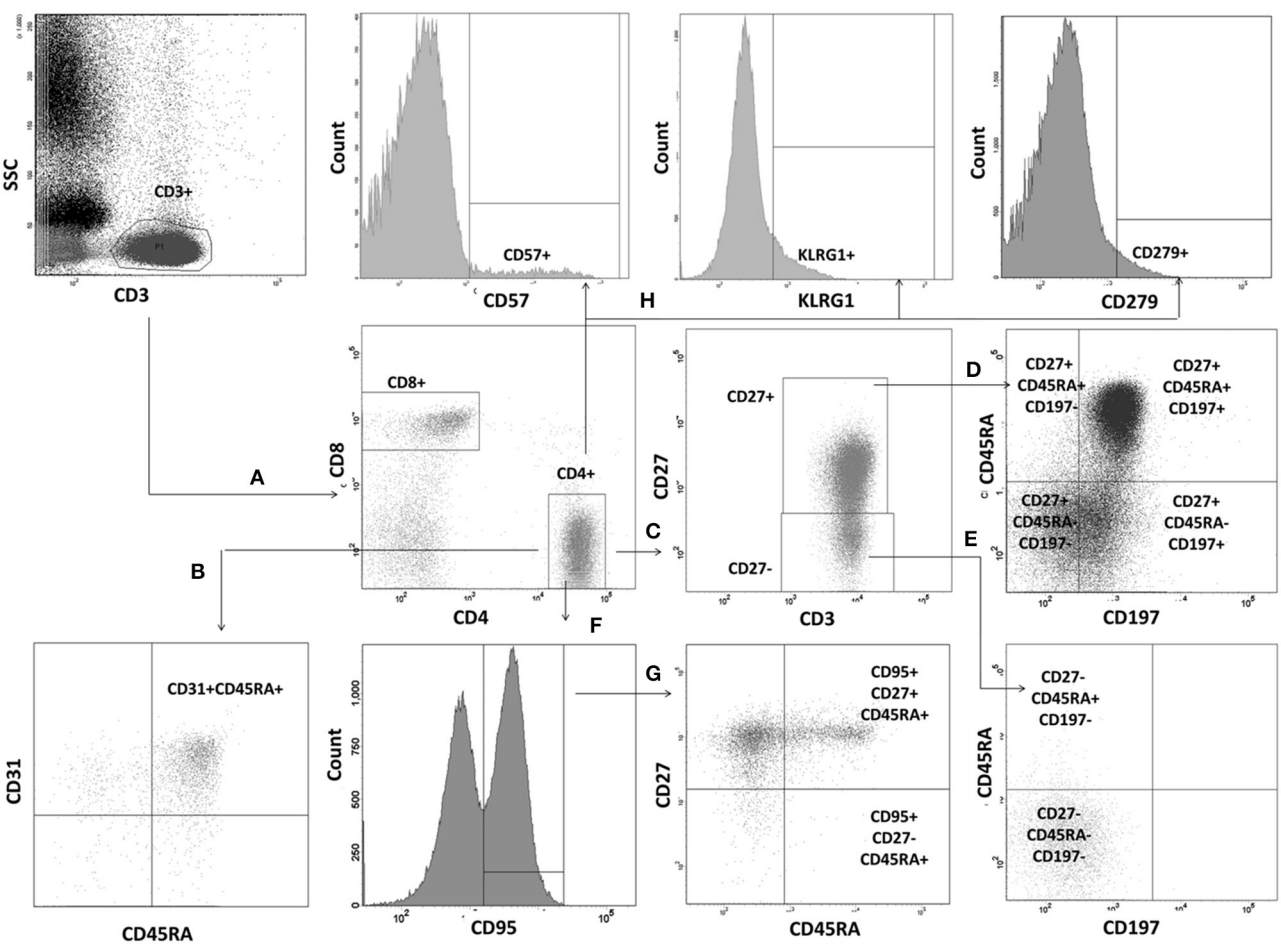
Gating strategy for evaluation of T lymphocyte maturation process. Lymphocyte subsets were identified by differential expression of CD27, CD31, CD45RA, CD95, and CD197. T lymphocytes were gated based on CD3 vs. side scatter characteristics. Identification of individual populations is presented for T helper cells. Identical strategy was used for T CD8^+^ cell subsets. **(A)** T helper and T suppressor cells were identified within T cell gate as CD3^+^CD4^+^ and CD3^+^CD8^+^, respectively. **(B)** RTE CD31^+^CD45RA^+^ were identified within the CD3^+^CD4^+^ gate. **(C)** Two additional gates: CD27^+^ and CD27^−^ were set. **(D)** Within CD27^+^ gate TN have been defined as CD27^+^CD45RA^+^CD197^+^, TCM as CD27^+^CD45RA^−^CD197^+^, TEM as CD27^+^CD45RA^−^CD197^−^, and low-differentiated effector RA^+^ L-TEMRA as CD27^+^CD45RA^+^CD197^−^). **(E)** High differentiated effector RA+ (H-TEMRA CD27^−^CD45RA^+^CD197^−^) and terminally differentiated (TD, CD27^−^CD45RA^−^CD197^−^) have been identified within the CD27^−^ gate. **(F)** CD95 gate was drawn within the T CD4^+^ gate. **(G)** Two populations were identified within CD95^+^ gate: TSCM (CD27^+^CD45RA^+^CD95^+^), and effector RA^+^ (TEMRA, CD27^−^CD45RA^+^CD95^+^). **(H)** Identification of cells with positive expression of CD57, KLRG1, and PD1 on the whole T CD4^+^ population.

Patients with NBS demonstrated significantly lower proportion and absolute lymphocyte count in comparison to healthy controls (27.2 vs. 46.8%, *p* < 0.01, and 1,330 vs. 2,676 cells/μl, *p* < 0.01). T lymphocytes composed significantly lower proportion and absolute count in NBS patients than in healthy controls (52.7 vs. 72.9%, *p* < 0.01, and 667 vs. 1,949 cells/μl, *p* < 0.01). Similar observation was made for CD4^+^ (25.9 vs. 43.1%, *p* < 0.01, and 322 vs. 1,074 cells/μl, *p* < 0.01) and CD8+ T lymphocyte subsets (16.7 vs. 22.8%, *p* < 0.01, and 234 vs. 612 cells/μl, *p* < 0.01), as well as B lymphocytes (8.7 vs. 14.1%, *p* < 0.01, and 103 vs. 359 cells/μl, *p* < 0.01). In contrast, NK composed a significantly greater population in NBS patients than in healthy controls (32.3 vs. 9.7%, *p* < 0.01, and 388 vs. 236 cells/μl, *p* < 0.01; Figure 2).

**Figure 3:**
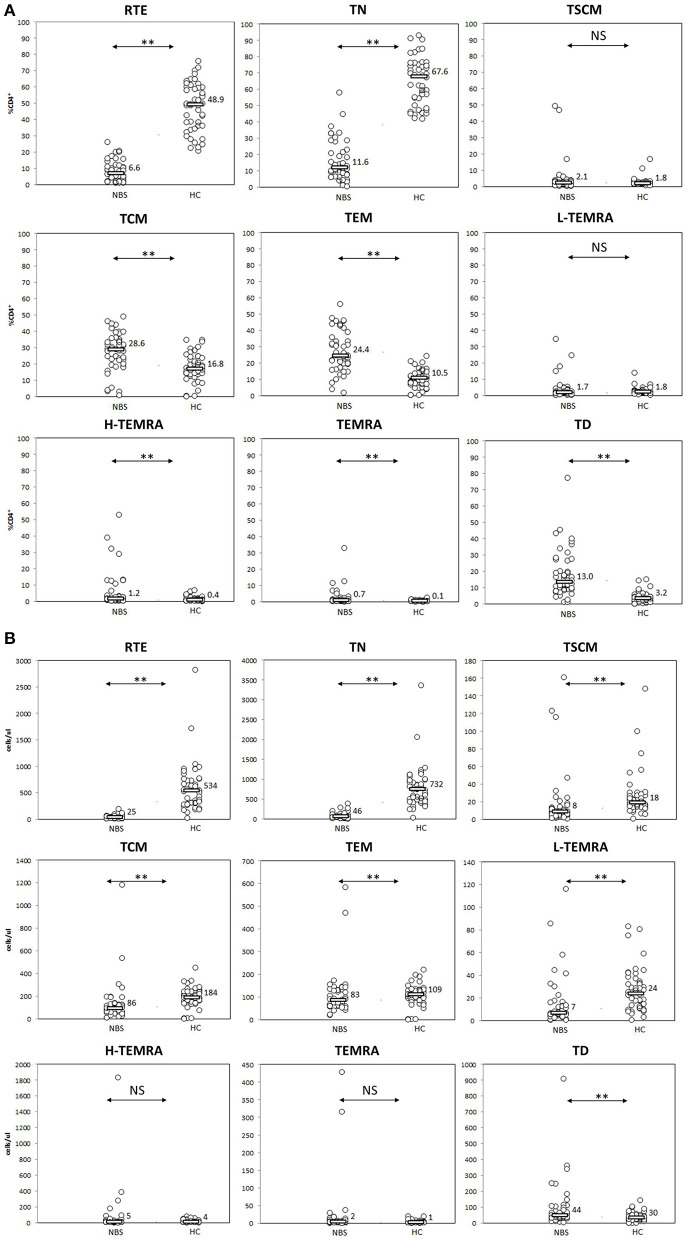
Peripheral T helper cell maturation was significantly disturbed. Individual results in respective study cohorts are presented as circles. Median values are presented as bars with numerical values. Statistical significance: NS, not significant, ** <0.01. **(A)** Median relative counts of T helper cell subsets in NBS patients in relation to normal control. Patients with NBS demonstrated significantly lower proportions of RTE and naïve helper cells, and significantly higher proportions of TCM, TEM, H-TEMRA, TEMRA, and TD lymphocytes. There was no statistical difference between TSCM and L-TEMRA. **(B)** Median absolute counts of T helper cell subsets in relation to normal control. Patients with NBS demonstrated significantly different absolute counts of all analyzed T helper subsets, except for H-TEMRA and TEMRA.

**Figure 4:**
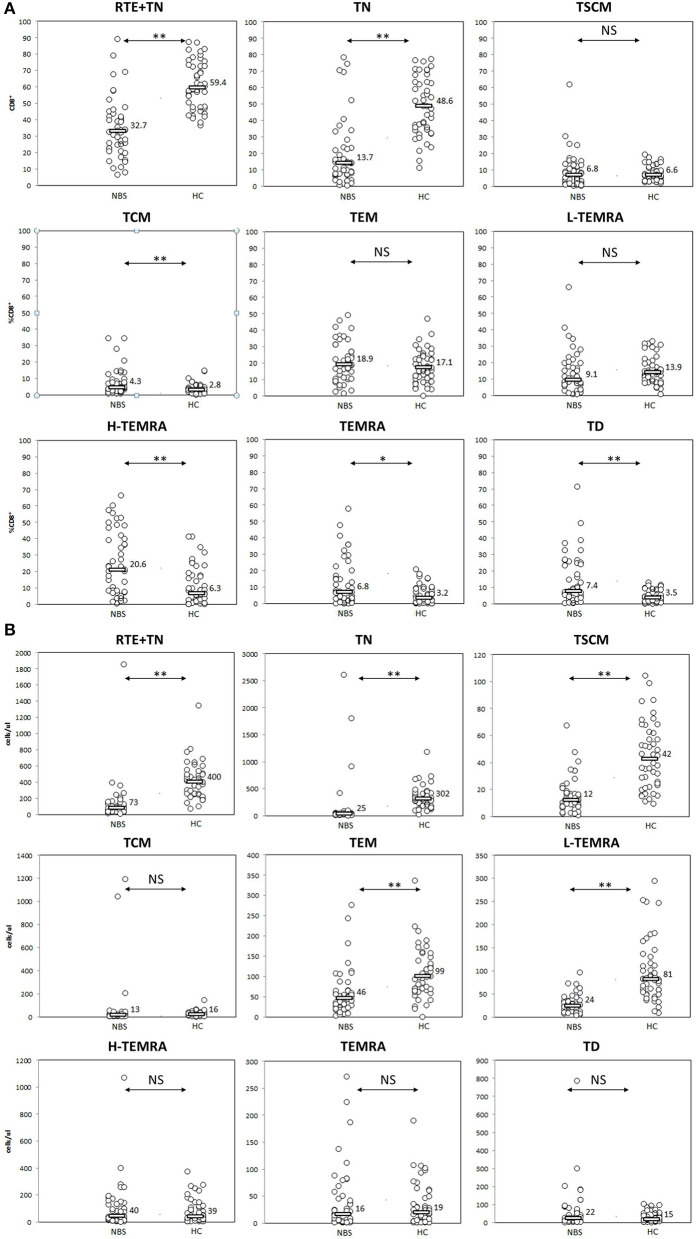
Peripheral T CD8^+^ lymphocyte maturation was significantly disturbed. Individual results in respective study cohorts are presented as circles. Median values are presented as bars with numerical values. Statistical significance: NS, not significant, * <0.05, ** <0.01. **(A)** Relative counts of T CD8^+^ cell subsets in NBS patients in relation to normal control. Patients with NBS demonstrated significantly lower proportions of T CD8 cells with phenotype corresponding to RTE (CD31^+^CD45RA^+^) and naïve cells, and significantly higher proportions of TCM, H-TEMRA, TEMRA, and TD lymphocytes. There was no statistical difference in relative distribution of TSCM, TEM, and L-TEMRA. **(B)** Absolute counts of individual studied CD8^+^ T lymphocyte populations. Significantly smaller populations of CD31^+^CD45RA^+^, TN, TSCM, TEM, and L-TEMRA cells were observed in NBS patients in comparison to controls. TEM, H-TEMRA, TEMRA, and TD composed similar populations in NBS and healthy cohorts.

We found several aberrancies in the T lymphocyte maturation profile in NBS patients in comparison to healthy controls, both in terms of relative and absolute counts of individual cell populations. Populations of lymphocytes identified as CD31^+^CD45RA^+^ and CD27^+^CD45RA^+^CD197^+^, were significantly less numerous in NBS patients than in healthy controls, both within the CD4^+^ (median 6.6 vs. 48.9%, 25 vs. 534 cells/μl *p* < 0.01, and 11.6 vs. 67.6%, 46 vs. 732 cells/μl, *p* < 0.01, respectively) and CD8^+^ cell subset (median 32.7 vs. 59.4%, 73 vs. 400 cells/μl, *p* < 0.01, and 13.7 vs. 48.6%, 25 vs. 302 cells/μl, *p* < 0.01, respectively; Figures 3, 4). Despite poor generation of naïve cells, we identified lack of differences in proportions of TSCM, considered to be the youngest antigen-primed T cell population, between NBS patients and healthy controls (median 2.1 vs. 1.8% CD4^+^, *p* = NS and 6.8 vs. 6.6% CD8^+^, *p* = NS), although significant differences in absolute counts of these cells were still detected both within CD4^+^ (8 vs. 18 cells/μl, *p* < 0.01), and CD8+ subset (12 vs. 42 cells/μl, *p* < 0.01). TCM composed significantly greater proportion of lymphocytes in NBS patients than in healthy controls (28.6 vs. 16.8% T CD4^+^, *p* < 0.01 and 4.3 vs. 2.8% T CD8^+^, *p* < 0.01), but significant differences in absolute counts were found only within the CD4^+^ cell subset (CD4^+^ 86 vs. 184 cells/μl, *p* < 0.01; CD8^+^ 13 vs. 16 cells/μl, *p* = NS). TEM composed a significantly greater population of T CD4^+^ in NBS patients (median 24.4 vs. 10.5%, *p* < 0.01, and 83 vs. 109 cells/μl, *p* < 0.01), with a similar population within T CD8^+^ lymphocytes (median 18.9 vs. 17.1%, *p* = NS), however, still significantly lower absolute number of cells from this population (46 vs. 99 cells/μl, *p* < 0.01). Low differentiated revertant CD45RA^+^ T lymphocytes composed similar populations within CD4^+^ and CD8^+^ cells in NBS patients in relation to healthy controls (median 1.7 vs. 1.8%, *p* = NS, and 9.1 vs. 13.9%, *p* = NS, respectively). On the other hand, more differentiated stages of TEMRA, i.e., H-TEMRA composed significantly higher proportions of T cells in NBS patients than in healthy controls (median 1.2 vs. 0.4% T CD4^+^, *p* < 0.01, and 20.6 vs. 6.3% T CD8^+^, *p* < 0.01). The same observation was made for TEMRA identified as CD27^−^CD45RA^+^CD95^+^ (median 0.7 vs. 0.1% T CD4^+^, *p* < 0.01, and 6.8 vs. 3.2% T CD8^+^, *p* < 0.05). In terms of absolute counts L-TEMRA were significantly less numerous within both CD4^+^ and CD8^+^ populations (7 vs. 24 cells/μl, *p* < 0.01, and 24 vs. 81 cells/μl, *p* < 0.01, respectively), but no significant differences were found either for H-TEMRA (5 vs. 4 cells/μl, *p* = NS and 40 vs. 39 cells/μl, *p* = NS, respectively) or total TEMRA identified as CD27-CD45RA+CD95+ (2 vs. 1 cells/μl, *p* = NS and 16 vs. 19 cell/μl, *p* = NS, respectively). The most differentiated stage of CD27^−^CD45RA^−^CD197^−^ T lymphocytes composed patients significantly greater proportion of both CD4^+^ and CD8^+^ cell subsets in NBS patients in comparison to healthy controls (median 13.0 vs. 3.2% T CD4^+^, *p* < 0.01, and 7.4 vs. 3.5% T CD8^+^, *p* < 0.01, respectively), but statistical difference in absolute count was found only in case of CD4+ cell subset (44 vs. 30 cells/μl, *p* < 0.01), with similar counts of TD cells within CD8+ subset (22 vs. 15 cells/μl, *p* = NS; Figures 3, 4).

**Figure 5:**
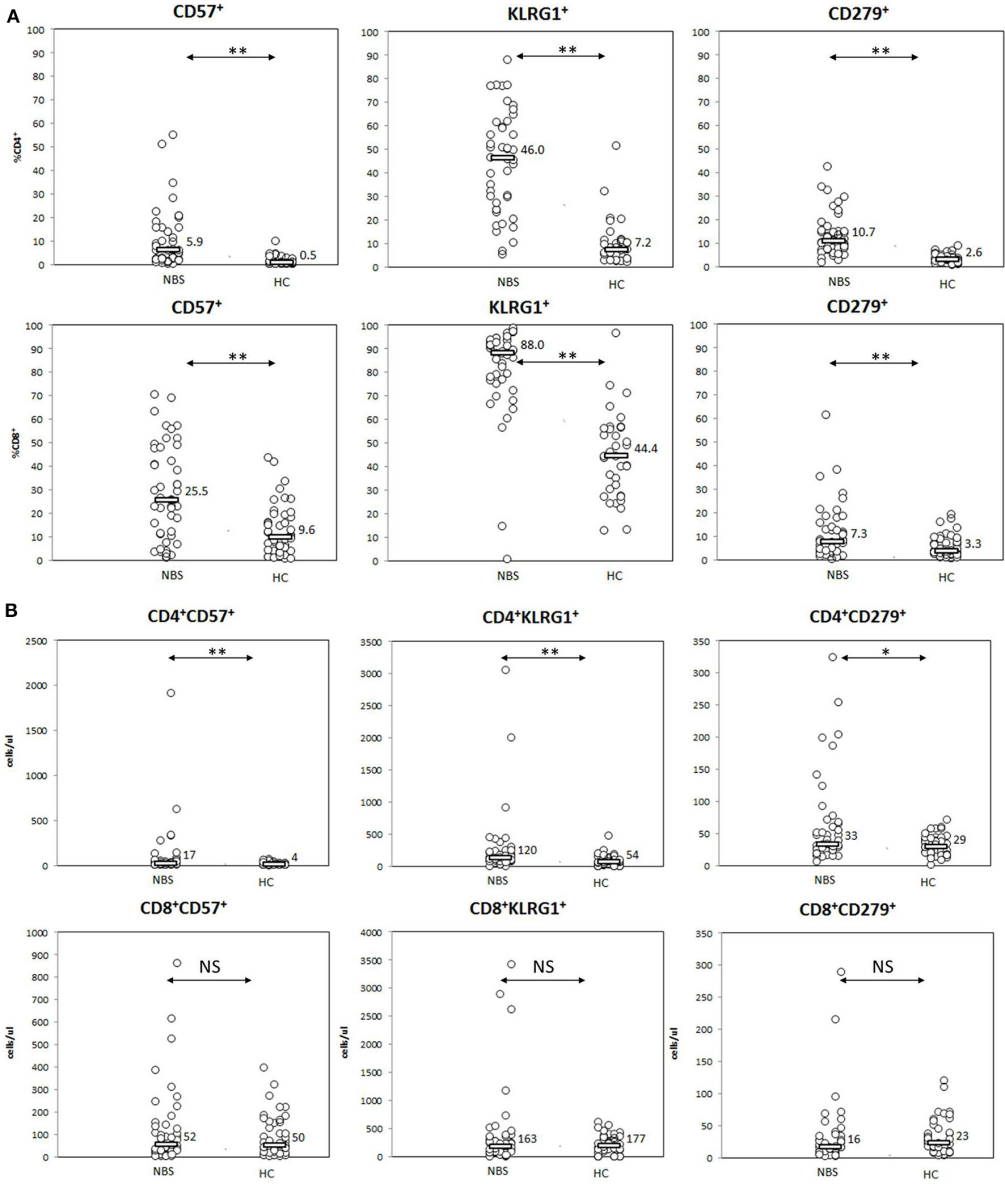
Expression of senescence (CD57, KLRG1) and senescence (PD1=CD279) cell markers. Statistical significance: NS, not significant, * <0.05, ** <0.01. **(A)** Patients with NBS demonstrated significantly elevated proportions of T lymphocytes (from both CD4^+^ and CD8^+^ subsets) with features of senescence (expression of CD57 and KLRG1), as well as exhaustion (CD279) than healthy controls. **(B)** T helper cells composed significantly more numerous populations of cells expressing (CD57, KLRG1, and CD279) in NBS patients than healthy controls. No statistical difference in absolute counts within the studied populations was found among T CD8^+^ lymphocytes.

Significant differences between NBS patients and healthy controls were found in expression of senescence markers CD57 and KLRG1, especially in terms of proportion of cells expressing the studied cell markers. CD57 was present on median 5.9 vs. 0.5% T CD4^+^ (*p* < 0.01) and 25.2 vs. 9.6% T CD8^+^cells (*p* < 0.01), with significant differences in absolute counts of CD57^+^ CD4^+^ cells (17 vs. 4 cells/μl, *p* < 0.01), but not within the CD8^+^ cell population (52 vs. 50 cells/μl, *p*= NS). Similar observation was made for KLRG1, which was detected on median 46.0 vs. 7.2% T CD4^+^ lymphocytes (*p* < 0.01) (120 vs. 54 cells/μl, *p* < 0.01) and 88.0 vs. 44.4% T CD8^+^ lymphocytes (*p* < 0.01). No statistically significant difference in absolute count of KLRG1^+^ CD8^+^ lymphocytes was found (163 vs. 177 cells/μl, *p* = NS; Figure 5).

PD1 as a marker of exhaustion was detected on a significantly higher proportion of both CD4^+^ and CD8^+^ lymphocytes in NBS patients than in healthy controls, with median CD4^+^PD1^+^ lymphocytes composing 10.7 vs. 2.6% (*p* < 0.01) and CD8^+^PD1^+^ composing 7.3 vs. 3.3% (*p* < 0.01). However, statistical differences in absolute counts between the study cohort and healthy controls were found only within CD4^+^ population (33 vs. 29 cells/μl, *p* = 0.0198), but not within CD8^+^ cells (16 vs. 23 cells/μl, *p* = NS; Figure 5).

## Discussion

This study for the first time presents a detailed analysis of T lymphocytes and their subpopulations that reflect the development of T cells in periphery in a large cohort of patients with Nijmegen breakage syndrome. Thus, our observations extend and supplement earlier information on T cell development in this rare, but still underestimated disease.

For many years CD45 isoforms were used to differentiate between naive (CD45RA^+^CD45RO^−^) and memory (CD45RA^−^CD45RO^+^) T lymphocytes (27, 28). Along with the development of more refined methods of cell analysis, it was found that other surface markers, such as CD197 corresponding to chemokine receptor CCR7 (29–31), CD27 (32–34), CD31 (35), and CD95 (36, 37), used in combination with CD45RA offered significantly more detailed view into the T lymphocyte maturation process (20). New combinations of markers allowed unique identification of recent thymic emigrants among T CD4^+^ cells (CD31^+^CD45RA^+^) (35, 38). In contrast to CD4^+^, the population of CD31^+^CD45RA^+^CD8^+^ T lymphocytes includes also a naïve subset (39, 40). Several newly identified populations, such as CD27^−^CD45RA^+^CD197^+^CD95^+^ (currently known as TEMRA) or CD27^+^CD45RA^+^ CD95^+^ (currently known as TSCM), have been initially identified among T CD8^+^ cells and only later among T CD4^+^ lymphocytes (29, 41). The newly identified cell subsets were also found to demonstrate varying effector capabilities (42–44) and significant differences in response depending on stimulating antigen (45–47).

Nibrin is known to participate in T cell development (48, 49) by affecting thymic output (50) and the V(D)J rearrangement process (6, 51, 52) resulting in increased proportions in T lymphocytes expressing TCRγδ receptor (19) and shift toward increased proportions of cells in mature development stages (14, 19, 53). The purpose of this prospective study was to provide more detailed description of the T lymphocyte maturation process in patients with Nijmegen Breakage Syndrome than available from previous reports (19, 53). We also searched for differences in the process between healthy subjects and patients with the DNA double-strand break repair defect caused by mutations in *NBN* gene.

The distribution of relative and absolute counts of basic lymphocyte subsets, i.e., T, B, NK, CD4^+^, and CD8^+^ T lymphocytes in the cohort under study did not differ from previous reports (7, 14, 15, 19, 50, 53). It was found that the naïve T lymphocytes subpopulation in NBS patients was significantly smaller in comparison to healthy controls, both within CD4^+^ and CD8^+^ cells, as expected in view of the previously reported reduced expression of CD45RA (19, 53). Our data demonstrated also, that thymic production of T helper lymphocytes measured by proportions and absolute counts of CD31^+^CD45RA^+^ cells was ineffective (50, 53) and resulting in significantly lower number of naïve T CD4^+^ cells in NBS patients than in control subjects (Figure 3). The population of T CD8^+^ lymphocytes described by CD31^+^CD45RA^+^ immunophenotype was significantly more numerous than of naïve CD8+ cells (Figure 4). Even though CD31^+^CD45RA^+^ within T CD8^+^ are not limited to recent thymic emigrants (40), significantly lower proportions of CD31^+^CD45RA^+^ and TN indicate that thymic production of naïve T CD8^+^ lymphocytes is significantly affected by the mutated variant of nibrin.

Unexpectedly, TSCM were found to compose similar proportions of CD4^+^ and CD8^+^, with significantly smaller absolute number of cells in NBS patients than in controls. Unaware of their existence, authors of previous reports included TSCM among naïve CD45RA^+^ cells, as sharing common phenotypic characteristics (CD27^+^CD45RA^+^CD45RO^−^CD197^+^) (19, 53). Their functional properties are however completely different, as TSCM are antigen-experienced, and they exhibit effector activity in contrast to quiescent naïve T lymphocytes (36, 54, 55). Therefore, it seems that despite low thymic production, T lymphocytes in patients with NBS have enough potential to differentiate from naïve into more mature TSCM, but it is not sufficient to overcome low thymic production. Yet, this increased proliferative potential in comparison to physiology, might in turn potentially lead to the increased susceptibility to malignancies of lymphoid origin observed in NBS patients (9, 10).

Homeostatic proliferation ensures the longevity of TCM in absence of cellular differentiation or activation. After proliferation, TCM can efficiently differentiate into effector cells (56). These processes seem not to be affected negatively by mutations in *NBN*, as TCM compose significantly greater proportion of both CD4^+^ and T CD8^+^ lymphocytes. TEM, which are potent effectors in healthy subjects, are also generated in greater proportions in NBS patients in comparison to healthy controls, but only within the CD4^+^ T lymphocyte population (Figure 3).

Similarly as in case of naïve cells, neither TCM, TEM, nor L-TEMRA within T CD4^+^ helper cells reached absolute counts observed in healthy controls. H-TEMRA and TEMRA composed similar populations, while TD CD4^+^ were even more numerous in NBS patients than in controls. Among CD8^+^ T lymphocytes, TEM, and L-TEMRA were significantly less numerous, while TCM, H-TEMRA, TEMRA, and TD reached similar absolute counts as in healthy controls (Figure 4). The explanation of important differences in maturation and proliferative potential between CD4+ and CD8+ lymphocytes reflected by the number of cells within individual populations requires more detailed molecular and functional analyses.

Preliminary data regarding age-related distribution of individual cell populations indicate that thymic output is deeply defective in almost all age groups. The defect seems to be more pronounced in younger children. In several patients, the absolute counts of all antigen primed CD4+ lymphocyte populations reached normal or almost normal counts. Adults appear to be an exception from this general observation, as recent thymic emigrants and naïve cells may reach low normal limits in some patients (Supplementary Figure 1A). This however does not mean that more cells are produced, as low limits of normal ranges in adults are lower than in young patients. Yet, more cells appear to be generated in several adults for antigen-primed populations. CD8+ T lymphocytes appear to behave differently in many terms (Supplementary Figure 1B). Deep defect in thymic production was observed in children and most adolescents, but not in adults. Children below 2 years of age seem to generate normal cell counts since reaching H-TEMRA maturation stage, while several patients from other age groups produce almost normal or normal counts of earlier antigen-primed cell populations. Moreover, several adolescents and adults seem to generate even higher than normal cell counts from the analyzed cell populations. These observations and their clinical context need to be verified in relation to larger group of healthy controls, when age related normal values will be established (study in progress).

Results of our study demonstrate that evaluation of the T lymphocyte maturation process in NBS patients by differential expression of only CD45RA/RO isoforms is misleading. Despite unsatisfactory thymic production, the generation of effector cells seemed quite effective and probably explaining relatively low incidence of clinical manifestations associated with cellular immune deficiency (17). Despite differences in biological properties, both TCM and TEM were correctly included in previous reports within the increased CD45RO^+^ population (19). The same applied to the terminally differentiated populations, which were generated in significantly greater proportions in NBS patients in comparison to controls. TEMRA, which develop in parallel with TD (57), represent a revertant population that re-express CD45RA, but also express CD45RO isoform. They must have been incorrectly included in both naïve and memory populations (19, 53). Therefore, previous reports overestimated both the population of naïve cells defined as CD45RA^+^, and memory population of CD45RO^+^ cells (19, 53).

Mutations in the *NBN* gene are known to be associated with the telomere-initiated senescence (58, 59). We analyzed the features of pre-term senescence of T lymphocytes in NBS patients by evaluating the expression of CD57 and KLRG1. Although CD57^+^ T lymphocytes are known to demonstrate cytotoxic abilities (49, 60, 61), the expression of CD57 is also associated with proliferative instability, correlating directly with the number of cell divisions and inversely with telomere length (43, 62). We found significantly increased proportions of CD57^+^ T lymphocytes both among CD4^+^ and CD8^+^ populations (Figure 5). This, however, could not be correlated (or solely correlated) to increased proportions of cytotoxic cells raised during viral infections, as no difference in CD57 expression between patients demonstrating chronic EBV viremia or EBV-free were observed (study in progress).

Surface expression of an inhibitory killer-cell lectin-like receptor G1 (KLRG1) identifies T lymphocytes that have undergone a large number of cell divisions (63). In healthy subjects, predominant expression of KLRG1 is observed on TEM and TEMRA cells (64). Both populations demonstrate potent effector functions, but are unable to proliferate (65). We found significantly increased proportions of KLRG1^+^ cells, corresponding to significantly increased proportions of TEM and TEMRA lymphocytes in patients from the study group (Figure 3). Similar suggestion regarding preterm T lymphocyte senescence in NBS was previously made by Meijerset al., based on results of studies performed in a significantly smaller group of patients (50). Therefore, considering the reported association of CD57 and KLRG1 with immune senescence (49, 66) and results of our flow cytometric experiments we conclude that patients from the study group demonstrate features of preterm senescence.

Functional impairment of T lymphocytes termed “exhaustion” is associated with an increased expression of PD1 (24, 66). We have observed significantly higher proportions of PD1^+^ T lymphocytes in NBS patients, both within T CD4^+^ and CD8^+^ subpopulations, which is in line with increased proportions of lymphocytes at the terminal differentiation stage. To our knowledge this feature has not been studied yet. Considering the features of an excessive proliferative history reflected by increased proportions of terminally differentiated and KLRG1^+^ T lymphocytes and increased proportions of PD1^+^ cells found in the study group in comparison to healthy controls, we conclude that T lymphocytes from NBS patients demonstrate features of exhaustion.

The difference between absolute counts of CD4^+^ and CD8^+^ lymphocytes demonstrating CD57, KLRG1, and PD1 expression appears to correspond with the observed differences in distribution and cell counts of the studied cell subsets (24, 67, 68). Significance of this discrepancy requires further analysis in context of clinical data.

We are aware of the limitations of the study. All experiments have been performed by flow cytometry and did not include either functional or molecular studies. Moreover, all references to functional properties of individual cell populations are based on published data. Such approach resulted mainly from limitations of the available research material, as large proportion of patients included minors, among them several below 2 years of age. Although functional properties of cells expressing CD57 or KLRG1 have not been evaluated in this study, premature senescence and shortened telomeres have been demonstrated *in vitro* in cultured *NBN*-mutated cells (69–71). Therefore, we feel the conclusion regarding premature senescence in NBS is justified.

In summary, we found significant aberration in peripheral T lymphocyte maturation process in NBS patients in comparison to physiological process. Despite low thymic production, the identified aberrancies and functional properties of individual T lymphocyte subpopulations lead to generation of significantly larger populations of effector T cells in NBS patients than in healthy people. Although this hypothesis needs further investigation, such properties might be related to an increased susceptibility to malignancy and milder clinical course than expected in view of T cell lymphopenia in patients with NBS.

## Data Availability Statement

The datasets generated for this study are available on request to the corresponding author.

## Ethics Statement

The studies involving human participants were reviewed and approved by Bioethical Committee at the Children's Memorial Health Institute, Warsaw, Poland. Written informed consent to participate in this study was provided by the participants' legal guardian/next of kin.

## Author Contributions

BP designed and supervised the flow cytometry experiments, analyzed and interpreted the data, and wrote the draft. BW-K designed the study, and collected and reviewed medical data. EH-P and AW collected and reviewed medical data. KT and UG performed the flow cytometry experiments. HG supervised the study. All authors critically revised and commented on the manuscript.

## Conflict of Interest

The authors declare that the research was conducted in the absence of any commercial or financial relationships that could be construed as a potential conflict of interest. The reviewer JD declared a past co-authorship with one of the author BW-K to the handling Editor.

## Supplementary Material

Supplementary Figure 1 Preliminary data regarding differences in age-related distribution of individual cell populations. Comparison of individual results of absolute counts of analyzed T lymphocyte populations between NBS patients (black circles) and healthy controls (white circles) in age-related groups **(A)**. Thymic output is deeply defective in almost all age groups, although it is more pronounced in younger children. In several patients, the absolute counts of all antigen primed CD4^+^ lymphocyte populations may reach normal or almost normal counts. Adults appear to be an exception from this general observation. Such phenomenon is observed already for recent thymic emigrants and naïve cells, which in some patients may reach lower normal limits **(B)**. CD8^+^ T lymphocytes appear to behave differently. Deep defect in thymic production is observed in children and most adolescents, but not in adults. Children below 2 years of age seem to generate normal counts since reaching H-TEMRA maturation stage, while many patients from other age groups produce almost normal or normal counts of antigen-primed cell populations. Several adolescents and adults seem to generate in several cases even higher than normal cell counts from the analyzed cell populations.

## Abbreviations

NBS: Nijmegen Breakage Syndrome
RTE: recent thymic emigrants
TN: naïve T lymphocytes
TSCM: memory T lymphocytes with stem-cell like properties
TCM: central memory T lymphocytes
TEM: effector memory T lymphocytes
TEMRA: revertant terminal effector memory T lymphocytes expressing CD45RA
L-TEMRA: low differentiated revertant terminal effector memory T lymphocytes expressing CD45RA
H-TEMRA: high differentiated revertant terminal effector memory T lymphocytes expressing CD45RA
TD: terminally differentiated T lymphocytes.
