# Supplementary figures and images for: T Lymphocytes in Patients With Nijmegen Breakage Syndrome Demonstrate Features of Exhaustion and Senescence in Flow Cytometric Evaluation of Maturation Pathway
Source: Front Immunol. 2020 Jun 30;11:1319. doi: 10.3389/fimmu.2020.01319 (PMC7338427; doi:10.3389/fimmu.2020.01319)

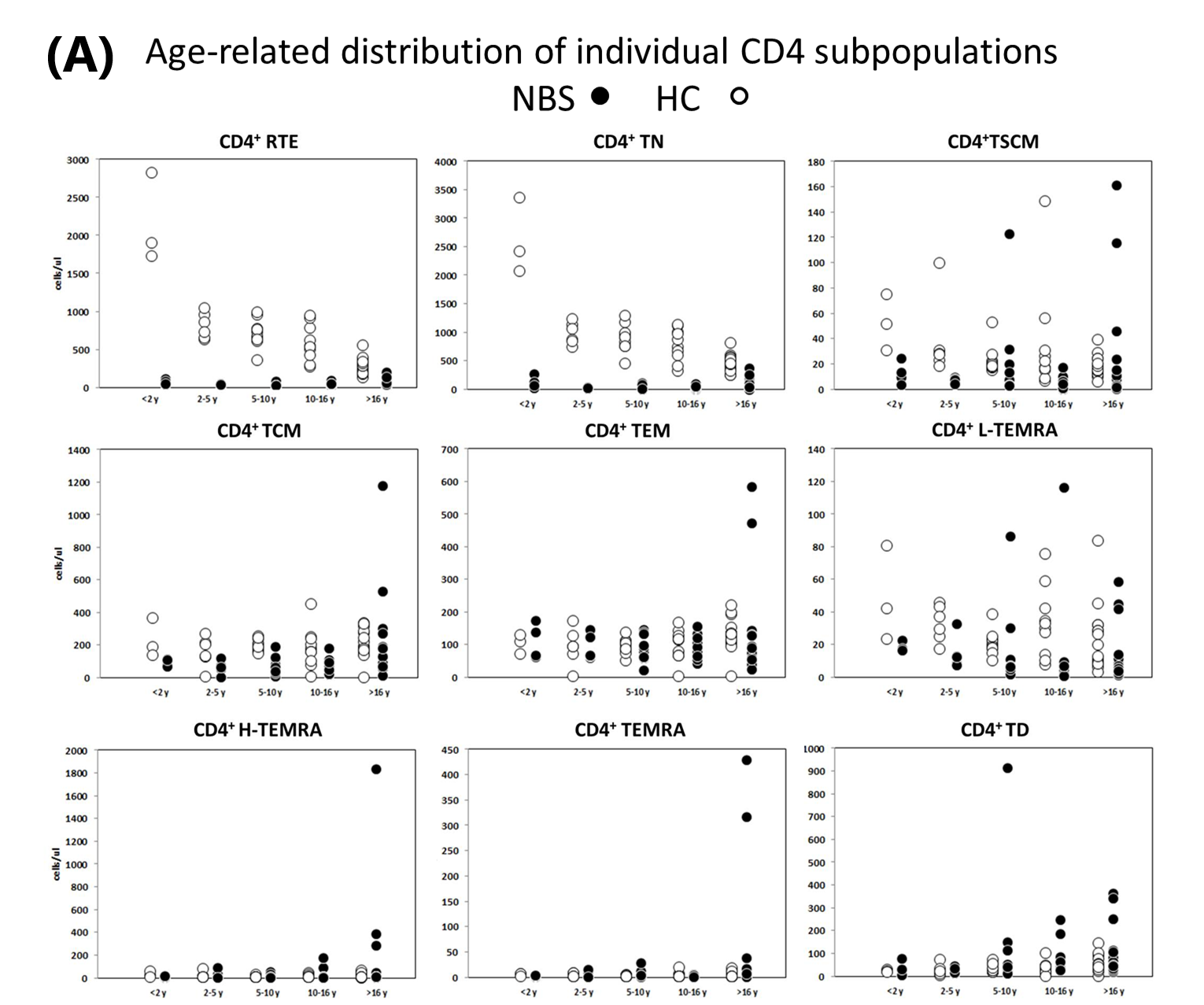

Supplement: Supplementary Figure 1 — Preliminary data regarding differences in age-related distribution of individual cell populations. Comparison of individual results of absolute counts of analyzed T lymphocyte populations between NBS patients (black circles) and healthy controls (white circles) in age-related groups (A). Thymic output is deeply defective in almost all age groups, although it is more pronounced in younger children. In several patients, the absolute counts of all antigen primed CD4+ lymphocyte populations may reach normal or almost normal counts. Adults appear to be an exception from this general observation. Such phenomenon is observed already for recent thymic emigrants and naïve cells, which in some patients may reach lower normal limits (B). CD8+ T lymphocytes appear to behave differently. Deep defect in thymic production is observed in children and most adolescents, but not in adults. Children below 2 years of age seem to generate normal counts since reaching H-TEMRA maturation stage, while many patients from other age groups produce almost normal or normal counts of antigen-primed cell populations. Several adolescents and adults seem to generate in several cases even higher than normal cell counts from the analyzed cell populations. [file Image_1.tiff]

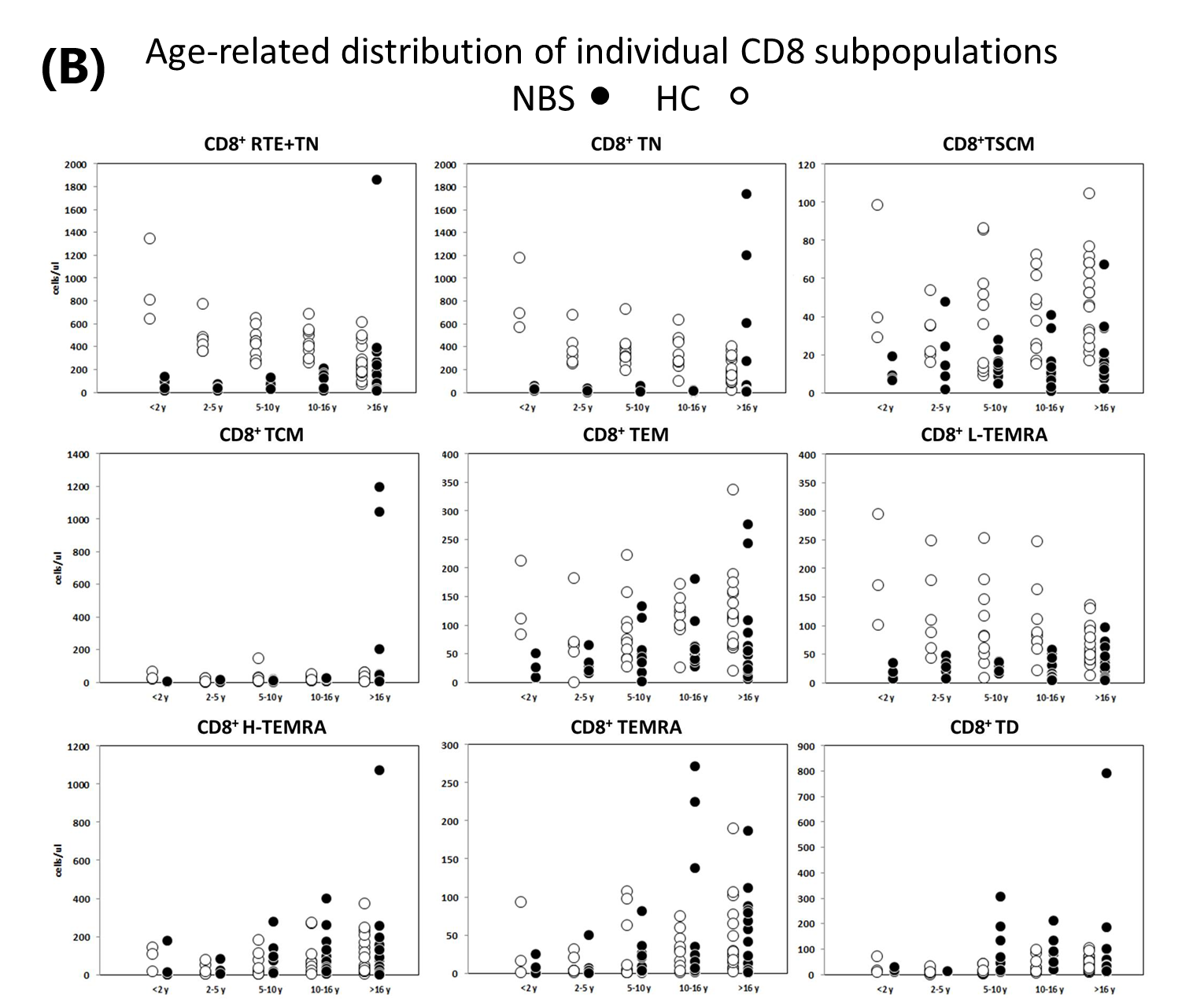

Supplement: Supplementary file 2 [file Image_2.tiff]
